# Structural Characterization of a Newly Identified Component of α-Carboxysomes: The AAA+ Domain Protein CsoCbbQ

**DOI:** 10.1038/srep16243

**Published:** 2015-11-05

**Authors:** Markus Sutter, Evan W. Roberts, Raul C. Gonzalez, Cassandra Bates, Salma Dawoud, Kimberly Landry, Gordon C. Cannon, Sabine Heinhorst, Cheryl A. Kerfeld

**Affiliations:** 1 MSU-DOE Plant Research Laboratory, Michigan State University, East Lansing, MI 48824, USA; 2 Physical Biosciences Division, Lawrence Berkeley National Laboratory, 1 Cyclotron Road, Berkeley, CA 94720, USA; 3 Department of Chemistry and Biochemistry, The University of Southern Mississippi, 118 College Dr. #5043, Hattiesburg, MS 39406, USA; 4 Department of Plant and Microbial Biology, UC Berkeley, Berkeley, CA 94720, USA; 5 Department of Biochemistry and Molecular Biology, Michigan State University, East Lansing, MI 48824, USA

## Abstract

Carboxysomes are bacterial microcompartments that enhance carbon fixation by concentrating ribulose-1,5-bisphosphate carboxylase/oxygenase (RuBisCO) and its substrate CO_2_ within a proteinaceous shell. They are found in all cyanobacteria, some purple photoautotrophs and many chemoautotrophic bacteria. Carboxysomes consist of a protein shell that encapsulates several hundred molecules of RuBisCO, and contain carbonic anhydrase and other accessory proteins. Genes coding for carboxysome shell components and the encapsulated proteins are typically found together in an operon. The α-carboxysome operon is embedded in a cluster of additional, conserved genes that are presumably related to its function. In many chemoautotrophs, products of the expanded carboxysome locus include CbbO and CbbQ, a member of the AAA+ domain superfamily. We bioinformatically identified subtypes of CbbQ proteins and show that their genes frequently co-occur with both Form IA and Form II RuBisCO. The α-carboxysome-associated ortholog, CsoCbbQ, from *Halothiobacillus neapolitanus* forms a hexamer in solution and hydrolyzes ATP. The crystal structure shows that CsoCbbQ is a hexamer of the typical AAA+ domain; the additional C-terminal domain, diagnostic of the CbbQ subfamily, structurally fills the inter-monomer gaps, resulting in a distinctly hexagonal shape. We show that CsoCbbQ interacts with CsoCbbO and is a component of the carboxysome shell, the first example of ATPase activity associated with a bacterial microcompartment.

The enzyme ribulose-1,5-bisphosphate carboxylase/oxygenase (RuBisCO) plays an essential role in the global carbon cycle by catalyzing the fixation of CO_2_ onto ribulose-1,5-bisphosphate and subsequent conversion of the intermediate to 3-phosphoglycerate (3-PGA) in the first step of the Calvin-Benson-Bassham (CBB) Cycle. This conversion is sufficiently slow to serve as the rate-limiting step of the CBB Cycle and, moreover, RuBisCO, is competitively inhibited by oxygen^1^,^2^,^3^. To compensate for the inefficiency of RuBisCO, cyanobacteria, some purple phototrophs and many chemoautotrophs have evolved a carbon concentrating mechanism that involves transporters to elevate cytosolic inorganic carbon levels and carboxysomes, bacterial microcompartments (BMCs) that sequester RuBisCO within a protein shell^4^,^5^,^6^,^7^,^8^. Cytosolic bicarbonate diffuses into the carboxysome where it is converted into CO_2_ by a resident carbonic anhydrase; this effectively elevates the substrate concentration near the active site of RuBisCO. The proteinaceous shell is thought to limit the loss of CO_2_^9^ and may restrict access of inhibitory O_2_.

Two types of carboxysomes have been classified based on the type of RuBisCO they encapsulate: Form IA RuBisCO in α-carboxysomes and Form IB RuBisCO in β-carboxysomes^10^. The model organism for α-carboxysome research is the sulfur-oxidizing bacterium *Halothiobacillus neapolitanus* (*H. nea*) because it is genetically tractable and because *H. nea* carboxysomes can be purified in high yield. Genes encoding α-carboxysome components constitute an operon, the *cso* operon^11^,^12^, which in *H. nea* includes the genes for the Form IA RuBisCO large and small subunits (*cbbL* and *cbbS*), the large shell protein CsoS2 that is essential for carboxysome assembly (*csoS2*)^13^, the shell-associated β-carbonic anhydrase CsoSCA (*csoS3*)^14^, the pentameric CsoS4A and CsoS4B proteins that form the vertices of the carboxysome shell (*csoS4A* and *csoS4B*)^15^,^16^, and the hexameric shell subunit assemblies of CsoS1A, CsoS1B, CsoS1C (*csoS1C*, *csoS1A*, and *csoS1B*) (Fig. 1a)^12^.

Recently structural and biochemical evidence demonstrated the presence of a pseudohexameric shell protein, CsoS1D, in the carboxysomes of the marine cyanobacterium *Prochlorococcus marinus* MED4^17^,^18^. This protein is encoded by a gene located upstream of the *cso* operon in α-cyanobacteria and downstream of the *cso* operon in *H. nea* (Fig. 1a), suggesting that it is likely differentially regulated^19^. Bioinformatic analysis has likewise expanded the locus of potential carboxysome-associated genes beyond the *cso* operon^13^,^18^. One of these, the pterin-4α-carbinolamine dehydratase-like (*pcd*-like) is absolutely conserved among all α-carboxysome gene clusters^18^. Its gene product was recently identified as a potential α-carboxysome RuBisCO assembly factor; it improved recombinant RuBisCO solubility when co-expressed with the GroEL/ES chaperonin complex in *E. coli*^20^. The need for such assembly factors for proper RuBisCO folding in plants and cyanobacteria is well documented; presumably they are required in chemoautotrophs as well^21^,^22^.

Another member of the expanded α-carboxysome locus is CbbQ, a member of the AAA+ superfamily proteins (ATPases Associated with diverse cellular Activities), which are found across all kingdoms of life and fulfill a variety of functions, mostly involved in ATP-driven dissociation, unfolding and remodeling of macromolecules^23^,^24^. Members of the superfamily share a structurally conserved ATPase domain of about 250 amino acids, which typically consists of a large N-terminal P-loop NTPase αβα domain and a smaller, all-α-helical, C-terminal domain. There are several key regions involved in ATP binding and hydrolysis which are highly conserved among all members of the family, including Walker A, Walker B, and arginine finger motifs. AAA+ proteins typically form cyclic hexamers in the presence of nucleotides and are active in this state. The nucleotide binding pocket is located between two subdomains and also at the interface between neighboring subunits. Hydrolysis and nucleotide release trigger conformational changes underlying the various functions.

One well characterized AAA+ domain associated with bacterial RuBisCO is RuBisCO activase (CbbX). CbbQ and CbbX (from *H. nea* and *Rhodobacter sphaeroides*, respectively) are only 18% identical, with the majority of homology in the AAA+ domain. In contrast to CbbX, there is relatively little experimental data available on the function of CbbQ. The CbbQ from *Hydrogenophilus thermoluteolus* was shown to have ATP binding and hydrolysis activity; it was estimated to be a decamer by size exclusion chromatography^25^. Orthologs from *Hydrogenophilus thermoluteolus* (formerly called *Pseudomonas hydrogenothermophila*) that are associated with noncarboxysomal form I RuBisCO, and two paralogs from *Hydrogenovibrio marinus* strain MH-110, associated with noncarboxysomal form I and form II RuBisCO, respectively, have been characterized. When they were co-expressed in *E. coli* with RuBisCO, there was a modest increase in RuBisCO activity^26^,^27^. Due to the difficulties of expressing functional RuBisCO in *E. coli*, which typically requires co-expression of chaperones, it is unclear whether this increase in activity would be the same in a native host. To date, the effect of CbbQ on the carboxysomal RuBisCO and on carboxysome function is unknown.

Here we report the characterization of the CbbQ ortholog that is associated with the α-carboxysome locus of *H. nea* (Hneap_0905). Bioinformatically we show that carboxysome locus-associated CbbQ (CsoCbbQ) orthologs form a distinct clade of CbbQ proteins. *H. nea* CsoCbbQ forms a complex when recombinantly co-expressed with CbbO, another member of the expanded *H. nea* α-carboxysome locus. We also determined the crystal structure of the *H. nea csocbbQ* gene product and confirmed ATPase activity in the recombinant protein. A deletion mutant in *H. nea* was generated to examine the potential role of CbbQ in carboxysome function. We found that CbbQ is tightly associated with the carboxysome shell, indicating that the structure and possibly the function of the carboxysome shell is more complex than previously thought. Our results suggest that a component of the α-carboxysome shell has ATPase activity.

## Results

### Bioinformatic Characterization of CbbQ Orthologs

We identified CbbQ orthologs in a variety of autotrophic bacteria that encode Form IA or Form II RuBisCO (Supplementary Fig. 1). The defining features are an N-terminal AAA+ domain (pfam07728) containing the characteristic residues and motifs for ATP binding, and a C-terminal domain confined to CbbQ members of the AAA+ superfamily (pfam08406). Phylogenetically, the CbbQ orthologs fall into four classes: those encoded proximal to the genes for 1) non-carboxysomal Form IA RuBisCO, 2) Form II RuBisCO, 3) the α-carboxysome superlocus (*csocbbQ*). The fourth class consisted of remote homologs not associated with RuBisCO or carboxysomes (Table 1). The *H. nea* genome encodes a carboxysome-associated and a Form II RuBisCO-associated CbbQ. The primary structures of the two CbbQ paralogs in *H. nea* are 71% identical. They are, however, less closely related to each other than to their orthologs from comparable genetic contexts (i.e. carboxysomal locus-associated or Form II-associated). The Form II-associated CbbQs form a distinct clade separate from the ones associated with carboxysomal and non-carboxysomal Form I RuBisCO (Supplementary Fig. 1, blue). Several CbbQ homologs in carboxysome-containing organisms are not found in any of those categories and form a separate clade (with the exception of Thimo_0165, which falls into the Form I RuBisCO-associated clade (Supplementary Fig. 1, grey). There is some overlap between non-carboxysomal Form IA-associated CbbQs and those associated with the carboxysome (Supplementary Fig. 1). Collectively these data implicate CbbQ in the biosynthesis, multimeric assembly or activation of the large subunit of RuBisCO.

**Figure 1:**
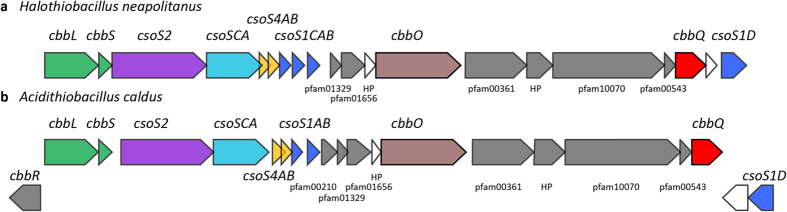
Gene neighborhood of carboxysome-associated ***cbbQ*** in ***H. nea*** (**A**) and (**B**). the representative α-carboxysome locus^28^ **encoded in the genome of** ***Acidithiobacillus caldus***. White colored genes are not conserved among loci^28^.

Recently, representatives of all distinct types of BMC loci encoded in bacterial genomes were described^28^. These loci frequently encode not only the proteins forming the microcompartment, but also transporters, regulators and other gene products that support the function of the BMC. The α-carboxysome locus of *Acidithiobacillus caldus* typifies that found in chemoautotrophs (Fig. 1b). The α-carboxysome locus of *H. nea* is organized slightly differently, but contains all of the expanded α-carboxysome locus components. The *csocbbQ* gene in *H. nea* (Hneap_0905) is separated from the terminal gene of the cluster, *csoS1D* by a gene for a hypothetical protein that is not conserved among loci (Fig. 1a). Between *csocbbQ* and the canonical carboxysome operon in *H. nea* there are several genes that are typically conserved in α-carboxysome loci; these include the gene for a pcd-like protein (pfam01329), and genes annotated as homologs of *parF*/Cbi (pfam01656), *cbbO*, *nuoL* (pfam00361), DUF2309 (pfam10070) and NPII/SbtB (pfam00543). CbbO genes are almost always encoded with *csocbbQ* (Supplementary Table 1, the only exception being A39ODRAFT_01696 from *Lamprocystis purpurea* DSM 4197), independent of their proximity to other proteins like RuBisCO, suggesting their functions are most likely connected^28^. Further support for a possible functional interaction between CbbQ and CbbO comes from the observation that von Willebrand Factor A (VWA) domain-containing proteins (such as CbbO) are frequently interaction partners with MoxR-like AAA+ domains, which include CbbQ^29^,^30^.

### X-ray Crystal Structure Determination

Hexa-histidine-tagged *H. nea* CsoCbbQ (Hneap_0905) was purified by affinity chromatography and crystallized with ATP in the mother liquor by vapor diffusion in sitting drops. Native data diffracting to 2.8 Å and belonging to space group H3 were collected (Table 2). Despite the presumed high structural homology of the AAA+ domain, molecular replacement using models based on related proteins (such as PDB IDs 2R44, 3NBX and 4AKG and other, more remote homologs) as search models did not result in any solutions of sufficient quality for model building, possibly due to the C-terminal domain of CbbQ that is not found in any available search model. Using a mercury derivative we obtained phases which enabled us to build an atomic model into calculated density of the isomorphous native dataset. We refined the structure to R_work_/R_free_ of 22.1/28.0% and 95% of the residues in the favored region of the Ramachandran plot (Table 2).

**Figure 2:**
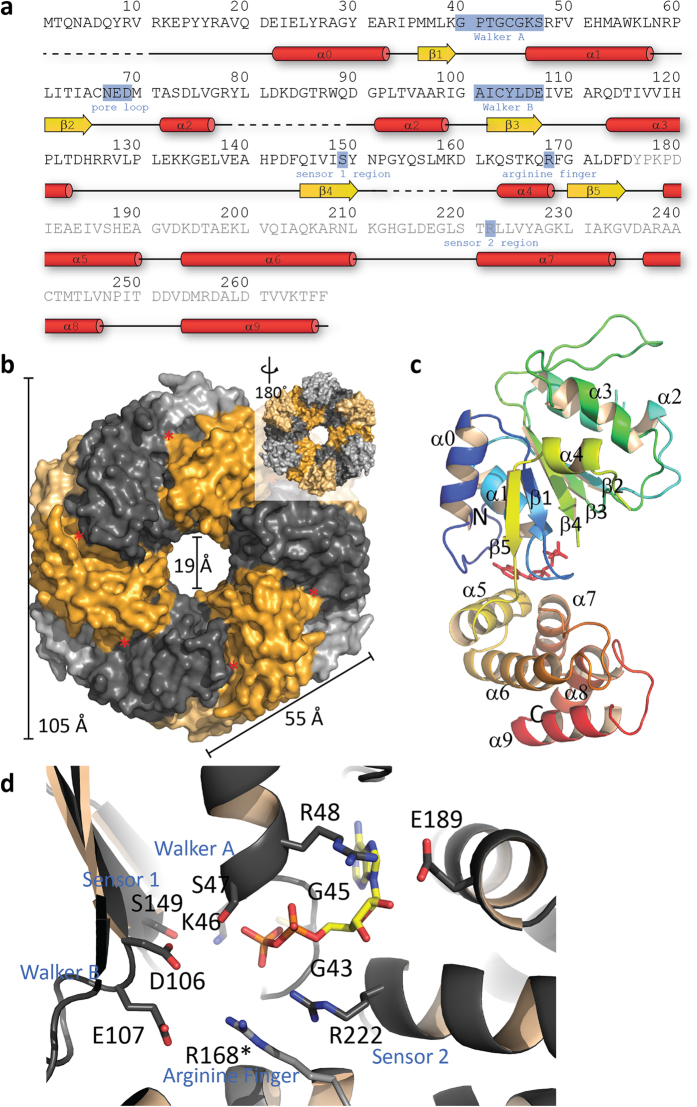
Structural overview of CsoCbbQ. (**a**) Primary and secondary structure of *H. nea* CsoCbbQ. Labeling of the helices and sheets according to standard nomenclature for AAA+ proteins. Red tubes indicate α-helices, yellow arrows β-strands and dashed lines residues disordered in the CsoCbbQ structure. The C-terminal domain is indicated by grey letters. (**b**) Surface representation of the CsoCbbQ hexamer with alternating monomers shown in gold and grey and N- and C-terminal domains colored in darker and lighter tone, respectively. Inset shows the reverse side. The red asterisk indicates the location of the nucleotide binding site, which is not accessible to the surface (**c**) Cartoon representation of CsoCbbQ monomer rainbow colored from blue (N-) to red (C-) terminus; ADP is shown as red sticks. (**d**) Close-up of the nucleotide binding site with relevant residues labeled and shown as sticks. Arginine finger residue R168* is contributed by the neighboring chain.

There are two CsoCbbQ molecules per asymmetric unit; expansion of crystal symmetry generates the full hexamer (Fig. 2b), consistent with the biologically active form of most AAA+ proteins and the size exclusion data (Supplementary Fig. 2). We were able to trace the protein backbone starting with residue R9 and, for one of the chains, extending to the C-terminus, with two loops missing between residues 79–89 and 153–159. One molecule of ADP is bound to each monomer, indicating that the ATP is hydrolyzed during crystallization. The two chains in the asymmetric unit align only moderately well (1.2 Å rmsd over all Cα atoms) with large differences observed in the C-terminal α-helices 8 and 9 (shifted by 3–4 Å) and with electron density for some loop regions missing in chain A. The differences observed could be explained by the inherent flexibility of the chains needed for the conformational changes coupled with ATP hydrolysis, which are typically transmitted to other proteins from AAA+ domain proteins.

Structurally, CsoCbbQ consists of the N-terminal P-loop NTPase domain (residues 1-175) characteristic of all AAA+ family proteins, comprising a beta sheet between two sets of α-helices (Fig. 2a,c). The order of the beta strands in the sheet is 5-1-4-3-2, typical for an AAA+ ATPase; α-helix 2 is interrupted by a partially disordered loop of 14 residues (G77-G91; Fig. 2a,c). The cyclic hexamer of N-terminal P-loop domains forms the central pore; the majority of inter-protomer contacts within the hexamer are formed by the NTPase domain. The N-terminal domain contains the characteristic Walker A (GXXXGK[T/S]) and Walker B (hhhhDE, h for hydrophobic residue) motifs (Fig. 2a,d) and is most closely related to the MoxR/dynein-related subfamily of AAA+ proteins (pfam07728). Sequence conservation is high throughout the entire NTPase domain (Supplementary Fig. 3) with the exception of the first 30 residues where only a PYY motif (residues 14–16) stands out. These tyrosine residues seem to be important for connecting the two subdomains; Y15 is involved in a π–π stacking interaction with the also highly conserved H188.

**Figure 3:**
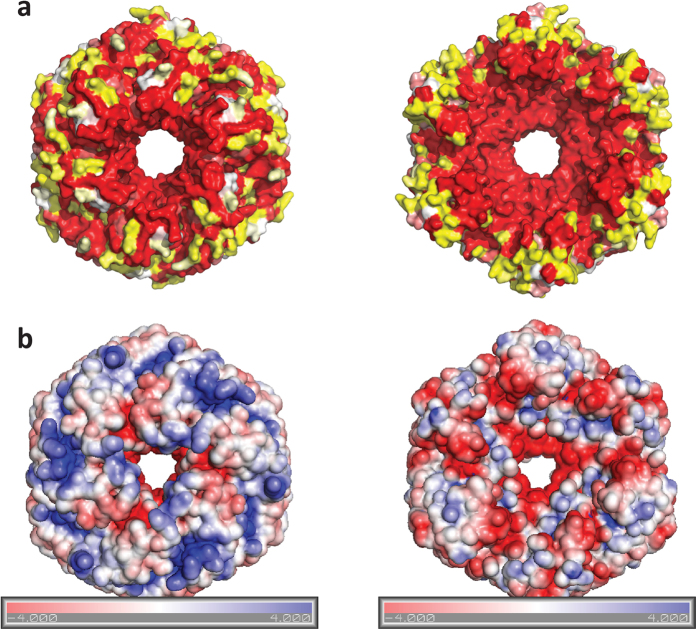
Surface representations (left convex, right concave, which includes the majority of the CbbQ specific domain) of (**a**) Sequence conservation mapped to the surface of CsoCbbQ (yellow low, white intermediate and red high conservation). (**b**) Electrostatics of the hexamer from both sides, coloring from −4 kT/e (red) to +4 kT/e (blue).

While the AAA+ domain of CbbQ can be structurally superimposed on its counterpart domain in CbbX, the C-terminal, CbbQ specific domain is structurally unique (Supplementary Fig. 4). The C-terminal domain (residues 176–270), the CbbQ-specific portion of the primary structure, forms a five-helix bundle that extends from the NTPase domain. (Fig. 2a,c). The CbbQ domain contributes only one residue, the conserved arginine finger residue R168, to the active site. Within the hexameric assembly, the majority of the CbbQ-specific domain is found on one surface of the hexamer (Fig. 2b), as a result this face of the hexamer is concave. Overall, the structure resembles a hexagon 105 Å in diameter (Fig. 2b). The pronounced hexagonal profile of CsoCbbQ is distinct from that of other structurally characterized AAA+ family members, which typically display a star-shaped outline^31^. This hexagonal profile of CsoCbbQ is a result of the gaps in the hexamer formed by the NTPase domain being filled by the five-helix bundles of each protomer (Fig. 2b). The central pore of CsoCbbQ is similar in size (19 Å) and charge to those of other AAA+ domain family members (Fig. 3b). The overall shape and straight edges of CbbQ are reminiscent of the CsoS1 protein hexamers which form the facets of the carboxysome shell. However, due to the different edge length (55 Å whereas in CsoS1A, a hexameric shell protein, the edges are 36 Å), it is not possible to readily fit CsoCbbQ into current models for the icosahedral carboxysome shell^32^.

### CsoCbbQ is a component of the H. nea carboxysome

**Figure 4:**
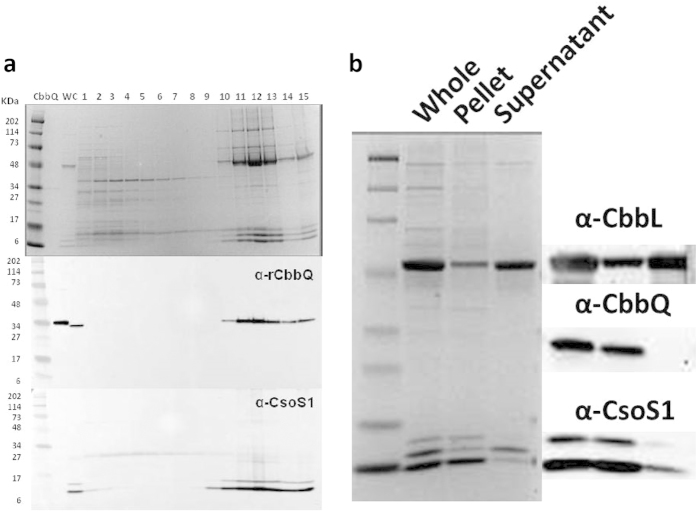
CsoCbbQ is associated with the carboxysome (**a**) Immunoblots reveal co-migration of CsoCbbQ with the major carboxysome shell protein CsoS1 during sucrose gradient centrifugation. CbbQ = purified, hexa-histidine-tagged recombinant CbbQ, WC = wild type carboxysomes, 1–15 = sucrose gradient fractions from top (#1) to bottom (#15) of the gradient; (**b**) Immunoblot of purified carboxysomes (Whole); following breakage by freeze-thawing, the presence of CbbQ in the shell enriched (Pellet) and absence from the released protein fraction (Supernatant) suggests that CsoCbbQ interacts strongly with the shell.

The occurrence of *cbbQ* in many chemoautotrophic α-carboxysome loci (Table 1) suggested a link between the CsoCbbQ paralog and the carboxysome. To investigate a potential physical association, purified *H. nea* carboxysomes were probed for the presence of CsoCbbQ. Co-migration of CsoCbbQ and the major carboxysome shell protein CsoS1 was investigated by immunoblotting^18^. The migration patterns of carboxysome-associated components can be monitored when sucrose gradient fractions of purified carboxysomes are loaded sequentially onto an SDS-PAGE gel and immunoblotted. Non-associated proteins sediment more slowly than carboxysomes during centrifugation, as shown in the gel image (Fig. 4a). The strongest CsoCbbQ and CsoS1 immunoblot signals are found in the same sucrose gradient fraction (#12), indicating CbbQ is associated with the *H. nea* carboxysome (Fig. 4a).

To further define the interaction between CsoCbbQ and the carboxysome, isolated carboxysomes were subjected to a freeze/thaw cycle known to disrupt the *H. nea* carboxysome while keeping the shells largely intact^16^. Following centrifugation, fractured carboxysomes are separated into a shell-enriched pellet and a protein-enriched supernatant that consists mainly of released internal RuBisCO molecules. As seen in Fig. 4b, the major shell protein CsoS1 is found in the shell-enriched pellet, while RuBisCO immunoblots reveal that most of the RuBisCO protein is released into the supernatant (Fig. 4b). The presence of an anti-CsoCbbQ signal exclusively in the shell-enriched pellet fraction indicates CsoCbbQ is associated with the carboxysome shell (Fig. 4b).

### Functional Characterization of CsoCbbQ

We established that CsoCbbQ has the ATPase activity predicted by the primary structure and from our crystal structure by spectrophotometrically measuring the production of ADP using a coupled assay with pyruvate kinase and lactate dehydrogenase^33^. CsoCbbQ demonstrated an average specific ATPase activity of 0.03 μmol ATP/min/mg CsoCbbQ at four different concentrations of CsoCbbQ (Supplementary Fig. 5). To determine if CsoCbbQ could function as a RuBisCO activase, the assay confirming the function of the red-type RuBisCO activase CbbX was undertaken with recombinant CsoCbbQ. We were unable to restore activity to *H. nea* RuBisCO inactivated by RubP (data not shown)^34^.

### CsoCbbQ forms a complex with CsoCbbO

**Figure 5:**
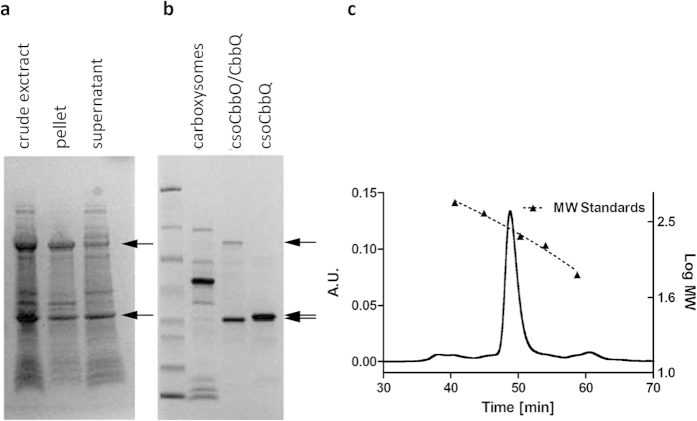
CsoCbbO/Q co-expression (**a**) Solubility analysis of the IPTG-induced co-expression of CsoCbbQ (bottom arrows) and N-terminal histidine-tagged CsoCbbO (top arrows) (**b**) hisCsoCbbO was soluble when co-expressed with CsoCbbQ and could be purified as a CsoCbbO/Q complex using the affinity tag on CbbO. The difference in molecular weight between csoCbbQ in the complex and individually expressed recombinant CsoCbbQ is due to the absence of the His_6_-tag on CsoCbbQ in the complex. (**c**) SEC revealed the complex elutes as a single peak at approximately 254 kDa. Molecular weight standards used were thyroglobulin (669 kDa), apoferritin (443 kDa), β-amylase (200 kDa), alcohol dehydrogenase (150 kDa), and BSA (66 kDa).

Based on the observation that CsoCbbQ and CbbO co-occur in a majority of non-cyanobacterial α-carboxysome loci, and that MoxR AAA+ domain proteins such as CbbQ typically interact with proteins that contain VWA domains (as in CbbO), we investigated the potential for interaction between CsoCbbQ and the cso-associated CbbO (CsoCbbO). CsoCbbO proved insoluble when expressed in *E. coli* under a variety of conditions. However, when non-His_6_-tagged CsoCbbQ (NT-CbbQ) and His_6_-tagged csoCbbO were co-expressed, we were able to purify a complex (Fig. 5a,b). This complex eluted as a single peak in size-exclusion chromatography at approximately 254 kDa (Fig. 5c), most likely corresponding to one CsoCbbQ hexamer and one CsoCbbO subunit. The complex shows ATPase activity comparable to that of recombinant CsoCbbQ.

### Characterization of HncbbQ::Km mutant

**Figure 6:**
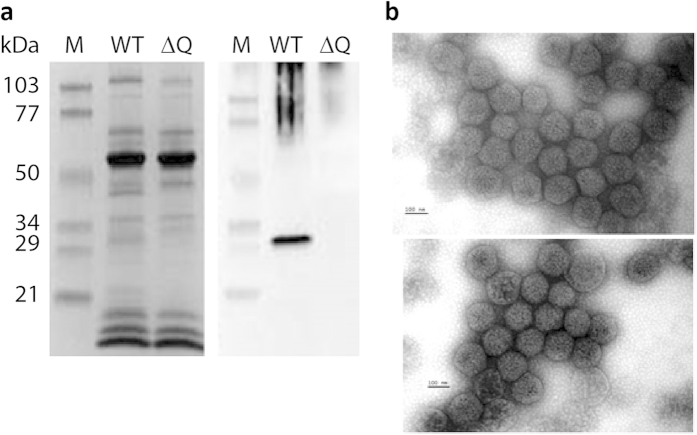
(**a**) Immunoblots of WT and mutant (ΔQ) carboxysomes show the mutant carboxysomes do not contain CsoCbbQ while the overall polypeptide composition is unaffected. (**b**) WT (top) and *csocbbQ::Km* (bottom) carboxysomes are virtually indistinguishable by TEM. Scale bar is 100 nm.

To further attempt to identify the role of CsoCbbQ in carboxysome function, we generated a *csocbbQ::Km* knockout mutant. The endogenous carboxysomal *csocbbQ* gene was replaced by a kanamycin resistance cassette using homologous recombination as previously described^35^. This insertion was confirmed by PCR and genomic DNA sequencing. Mutant cells did not produce CsoCbbQ, as confirmed by immunoblotting of purified mutant carboxysomes (Fig. 6a).

Carboxysomes were purified from the *csocbbQ::Km* strain and examined by TEM (Fig. 6b). Structurally important carboxysome components can produce irregularly shaped carboxysomes when deleted^36^,^37^. However, wild type (WT) and mutant carboxysomes were of similar shape and diameter, indicating that, as previously observed for other low-abundance components^15^,^16^,^36^, csoCbbQ is not a crucial structural component. There was also no detectable phenotype as monitored by measuring the OD_600_ of batch cultures of WT and *csocbbQ::Km* grown in air and in 5% CO_2_, respectively, over a period of 40 hours (Supplementary Fig. 6). Mutant cells displayed no observable growth limitations, compared to the WT, when grown in air. Elimination of CsoCbbQ did not affect the size, number or spatial organization of carboxysomes; the ultrastructure of mutant cells was comparable to wildtype under both low and high CO_2_ growth conditions as judged by transmission electron microscopy (data not shown).

## Discussion

We have determined the structure of CsoCbbQ, a AAA+ ATPase protein in its hexameric, nucleotide-bound form and show that it is an active ATPase. The occurrence of *cbbQ* near genes for Form I and Form II RuBisCO and prior evidence of its potential to affect the activity of recombinant RuBisCO in *E. coli*^27^,^38^ initially suggested two potential ATPase-associated functions for CsoCbbQ in the carboxysome: as an RbcX-like chaperone activity associated with RubisCO assembly and packaging or as an Rca (RuBisCO activase)/CbbX-like RuBisCO activase. In β-cyanobacteria, RuBisCO large and small subunit genes are frequently localized in an *rbcLXS* cluster with the Form I RuBisCO chaperone RbcX. This protein assembles as a homodimer that binds and stabilizes RbcL subunits during formation of the L8 core^39^. Inactivation of the *rbcX* gene in *Synechococcus* sp. 7002 resulted in a significant reduction in the amount of RbcL and RbcS subunits produced *in vivo*, while co-expression of RuBisCO subunits with RbcX in *E. coli* increased soluble hexadecamer formation and activity^40^. Such an effect on carboxysomal RuBisCO assembly in *H. nea* would be expected to yield reduced RuBisCO content in isolated mutant carboxysomes and likely a high CO_2_-requiring (*hcr*) phenotype in air-grown mutant cells, but no change in carboxysome polypeptide composition and morphology or growth rate and subcellular ultrastructure was observed in the *csocbbQ* mutant. However, the lack of a phenotype under “normal” growth conditions does not preclude a role for CsoCbbQ in RuBisCO assembly under specific (stress) conditions *in vivo*. No change in growth or RuBisCO content was observed in *Synechococcus* sp. PCC7942 *rbcX::Km*^*R*^ mutants, despite the fact that the RbcX gene product was shown to positively affect RuBisCO assembly in *E. coli*^41^.

The second prospective ATPase related function for CsoCbbQ, as a RuBisCO activase, likewise could not be confirmed. There is a complete lack of structural homology between the C-terminal domains of CbbX and CsoCbbQ, suggesting the two proteins have distinct functions (Supplementary Fig. 4). CsoCbbQ does have relatively modest ATPase activity - approximately 30-50x lower than the reported *in vitro* rate for Rca from *Arabidopsis* (1.0–1.5 μmol ATP/min/mg), 20x lower than the red-type RuBisCO activase CbbX from *Rhodobacter sphaeroides*^33^,^34^ and 40× lower than the CbbQ from *Hydrogenophilus thermoluteolus*^25^. CsoCbbQ ATPase activity might be higher with its cognate substrate, which is not known at present. We were unable to determine a potential *in vivo* rate of ATP hydrolysis due to the low copy number of CsoCbbQ in isolated carboxysomes, but the observation of *in vitro* ATPase activity suggests the α-carboxysome may support an additional enzymatic reaction that could affect RuBisCO activation.

Attempts to determine the function of CsoCbbQ are complicated by the observation that it complexes with CsoCbbO. The function of CsoCbbO is even more enigmatic than that of CsoCbbQ. It is a large (788 amino acids in *H. nea*) protein in which the only discernible feature within the primary structure is a VWA domain in its C-terminus, a domain frequently found in proteins that are associated with AAA+ proteins. When co-expressed in *E. coli,* the two proteins form a complex and CsoCbbQ renders CsoCbbO soluble. The role of an ATP-hydrolyzing molecular chaperone for CsoCbbO would appear to suit CsoCbbQ, but further characterization is required.

Deletion of *csocbbQ* did not impair growth or carboxysome morphology in *H. nea.* However, there is a second *cbbQ* gene associated with that encoding Form II RuBisCO in *H. nea*. The Form II RuBisCO of *Hydrogenovibrio marinus* is expressed at elevated CO_2_ concentrations and can compensate for a reduction in Form IA RuBisCO activity under those conditions^42^. The form II-associated CbbQ would also likely be expressed under such conditions; it is unknown if this CbbQ gene product is Form II-specific or if it could compensate for the loss of CsoCbbQ activity under any conditions.

The lack of a discernible effect on carboxysome ultrastructure in the *csocbbQ::Km* mutant may be related to its low abundance; deletion of other minor components from the alpha carboxysome likewise does not have a discernible effect on carboxysome morphology^15^,^16^,^36^. The copy number of CsoCbbQ in the shell may indeed be flexible and, moreover, likely requires identifying a triggering stress for its functional importance to manifest. Elevated CO_2_ was the only environmental condition altered during growth experiments, but it is possible that further physiological stresses are required for CsoCbbQ to exert an effect on RuBisCO in the carboxysome. The position of *csocbbQ* (as well as *csocbbO*) in the expanded locus, downstream of the canonical *cso* operon, suggests that it is likely independently regulated, perhaps as a response to stress or the availability of nitrogen.

CbbQ is a member of the MoxR family of ATPases, many of which function as chaperones^29^,^30^. Some MoxR ATPases are known to interact with a protein containing a VWA domain (pfam00092), as found in the C-terminal region of CsoCbbO. Recently, subunits of the NADH:Ubiquinone Oxidoreductase (Nuo) I complex were shown to be additional interaction partners for a MoxR ATPase and its cognate VWA protein^43^. NuoL homologs (pfam00662 and pfam00361) are conserved in the typical expanded α-carboxysome loci (Fig. 1b,^28^); they encode membrane-spanning domains that have been implicated in inorganic carbon uptake^44^. In *H. nea* the gene encoding NuoL is followed by a putative transmembrane hypothetical protein, a 1046 amino acid protein DUF2309 (pfam10070), a Nitrogen Regulatory protein PII/SbtB homolog, and a small hypothetical protein. The position of these genes between the verified interaction partners CsoCbbO and CsoCbbQ may indicate that their products play functional roles related to that of CsoCbbO and CsoCbbQ. In support of a functional relation among these gene products, the pfam00361 domain of NuoL is found in many proteins fused to pfam10070. Although speculative, in addition to co-expression, there may be physical interactions among these gene products of the expanded carboxysome locus that are important under some as yet to be identified conditions. This would account for the presumably different regulation of these genes relative to the canonical *cso* operon.

We have established that CsoCbbQ functions as an ATPase, as demonstrated both functionally (Supplementary Fig. 5) and structurally (Fig. 2d). Notably, the C-terminal domain (pfam08406) that is specific to CbbQ orthologs is a prominent feature of just one face of the hexamer (Fig. 2b). It consists of a bundle of five antiparallel helices, in contrast to most other AAA+ domain proteins that are composed of C-terminal four helix bundles. Structural homology searches using only the C-terminal domain result in only marginally scoring hits, with the best one being a ssRNA binding protein (PDB ID 2XC7) that has a sequence identity of 18% and an rmsd of 2.3 Å. This homology seems to be strictly structural though, since most of the residues binding the RNA are absent in CsoCbbQ. The highest conservation observed in the C-terminal domain is found where it is involved in intra- or inter-subunit contacts but also on the concave (CbbQ specific domain face) of the hexamer (Fig. 3a). Many of those residues are negatively charged (Fig. 3b). A possible explanation for this high conservation on the surface is that this region is important for interaction with a substrate or other protein(s), such as those forming the carboxysome shell or being part of a transmembrane carbon uptake complex.

The co-occurrence of CbbQ with RuBisCO in general and with the carboxysome in particular suggests a role possibly in either activation of RuBisCO or facilitating the packaging of RuBisCO into the α-carboxysome under some conditions. This is the first evidence for the association of an ATP-hydrolyzing enzyme with the carboxysome, as was speculated for the β-carboxysomes when Rca homologs that contained a domain putatively important for carboxysome localization were identified^45^. Additional biochemical and expression studies will be required to elucidate the precise function of CsoCbbQ; our negative results for activase activity may reflect the limitations of *in vitro* analysis outside of the confines of the carboxysome. The ATPase activity that we have identified and the novel AAA+ structure of CsoCbbQ will be valuable in interpretation of the results of further studies on CbbQ homologs.

Our results implicate CsoCbbQ as a component of the assembled carboxysome (Fig. 4a), where it is tightly associated with the shell (Fig. 4b). The striking hexagonal shape of the CsoCbbQ structure immediately evokes comparisons to carboxysome shell proteins, however, the CsoCbbQ hexamer is too large to be incorporated into existing models of carboxysome shell facets. The current working model of the carboxysome shell is based solely on structures of isolated shell protein hexamers (BMC-H) forming the facets, and vertices formed by pentameric BMC-P^32^ with the geometric constraint of icosahedral symmetry. Clearly, this model is too simplistic; it is becoming increasingly apparent that there are additional protein subunits integrated into or tightly associated with the carboxysome shell. The abundant CsoS2 protein is a crucial component of the α-carboxysome shell^13^,^46^, and CsoSCA is so tightly associated with the shell that it cannot be purified without disintegrating the shell^16^,^47^. While CsoCbbQ may be too large to be integrated directly into the CsoS1 hexamer shell layer, the matching symmetry could enable it to dock onto a hexagonal shell protein. The association of the ATPase CsoCbbQ adds additional complexity to our current model of the carboxysome shell and suggests that it functions as more than an inert semi-permeable barrier.

## Methods

### Cloning and Expression

The *csocbbQ* (Hneap_0905) gene was amplified via PCR from *H. neapolitanus* genomic DNA with forward primer 5′-*GGATCC*ATGACACAAAATGCAGATCAATATCG-3′ and reverse primer 5′-*AAGCTT*TTAAAAGAACGTTTTGACGACGG-3′ (italics represent added restriction enzyme sites). The amplified gene product was cloned into the pCR-BluntII-TOPO vector (Invitrogen) and sequenced. Positive clones were digested with restriction enzymes BamHI and HindIII (New England Biolabs) and ligated into linearized pCDFDuet-1 vector to give a pCDFDuet-csoCbbQ expression construct. IPTG-induced protein expression in chemically competent *E. coli* BL21(DE3) cells (Invitrogen) was carried out at 37 °C for 3 h, after which cells were lysed via three passes through a French Pressure cell. The crude extract was centrifuged, and soluble CsoCbbQ in the supernatant was purified via His_6_-tag on Ni^2+^/NTA affinity resin (Thermo Scientific). Purified CsoCbbQ was eluted with 250 mM imidazole and was dialyzed twice in 4 l of 10 mM Tris-HCl, pH 8.0. Final protein concentrations were determined by BCA assay (Thermo Scientific/Pierce).

### Shell association and ATPase activity

Co-migration and shell-association analysis was performed as described previously with a polyclonal primary antibody, generated by Cocalico Biologicals Inc., raised using affinity-purified his-CsoCbbQ for polyclonal antibody generation in rabbits, using the company’s standard inoculation protocol^18^. The rate of ATP hydrolysis was determined by spectrophotometrically measuring the rate of ADP formation in a coupled assay with pyruvate kinase and lactate dehydrogenase^33^. The reaction mixture contained 50 mM Tris-HCl pH 8.0, 20 mM KCl, 5 mM MgCl_2_, 2.5 mM ATP, 1 mM phosphoenolpyruvate, 0.3 mM NADH, 12 U/mL pyruvate kinase, and 12 U/mL lactate dehydrogenase. Recombinant CbbQ protein was added at final concentrations of 0, 3.7, 7.4, 14.8, and 29.8 μM in a total reaction volume of 200 μL in a 96-well plate. The absorbance at 340 nm was measured in a Synergy 2 Multi-Mode plate reader (Bio-Tek) at 45 second time points for 15 minutes.

### Mutant generation and characterization

The well-established protocol for generating carboxysome deletion mutants in *H. nea* was followed to yield *csocbbQ::Km* mutant cells^35^. A kanamycin resistance cassette was amplified with forward primer 5′-ttaaatcagaaagacggctacatcgaccagaacaaggcaatttaaCCGGAATTGCCAGCTGGG-3′ and reverse primer 5′-ctggtgtgtttcatgttgtgcgtattacctgttggtacggaaggcTCAGAAGAACTCGTCAAGAAGGCGATAG-3′. The lower case sequences represent regions of homology immediately upstream (forward primer) and downstream (reverse primer) of the endogenous *csocbbQ* gene and upper case sequences anneal to the Km^r^ cassette. Concurrently, a *H. nea* DNA fragment that encompasses the *csocbbQ* gene with adjacent 500 base pairs upstream and downstream was amplified from genomic DNA with forward primer 5′-*GGATCC*ATCCGGAAACGGGCGATCG-3′ and reverse primer 5′-*AAGCTT*GCGATGATGTCGCAAGATAGG-3′. The resulting PCR product was cloned into the pCR-BluntII-TOPO vector (Invitrogen), and the sequence was verified. The *csoCbbQ*-500 region was then ligated into the BamHI and HindIII restriction sites of the pUC18 vector to yield pUC18-csoCbbQ500. Homologous recombination in *E. coli* DY330 cells was used to replace the *csocbbQ* gene in pUC18-csoCbbQ500 with the amplified Km^r^ cassette, utilizing the 45 bp of homology added during PCR. The resulting pUC18-CbbQ500-Km^r^ replacement construct was electroporated into exponentially growing *H. nea* cells as described previously for replacement of the endogenous *csocbbQ* gene with the kanamycin resistance cassette by homologous recombination *in vivo*^35^.

Successful replacement of the *csocbbQ* gene with the Km^r^ resistance cassette was confirmed by genomic DNA sequencing. A culture of mutant cells was maintained in a Multifors 1-L chemostat (Infors HT) with kanamycin-supplemented medium at 30°C. Carboxysomes were isolated from chemostat-grown cells as described previously, and the absence of CsoCbbQ in mutant carboxysomes was confirmed by immunoblotting^48^. Ultrastructure of isolated mutant carboxysomes was examined by transmission electron microscopy (TEM). A 1.0 mg/mL carboxysome sample was loaded onto a 300-mesh Formvar-coated copper grid and stained with 1% ammonium molybdate, pH 8.0. Images were acquired with a Zeiss 900 TEM with a Model 785 ES1000W Erlangshen CCD camera at 140,000× magnification. Growth of mutant cells in air and 5% CO_2_ at 30 °C was monitored by measuring the OD_600_ of 100 ml batch cultures grown in Kanamycin-free media at several time points over a period of 42 hours. Growth of WT cells under identical conditions was monitored as a control.

### Complex characterization

The Duet vector expression system (Novagen) was used to co-express His_6_-tagged CsoCbbO (HT-CbbO) and CsoCbbQ without an affinity tag (NT-CbbQ). A pCDFDuet-NTCbbQ expression construct was generated by PCR amplification with the forward primer 5′-*CCATGG*ATGACACAAAATGCAGATCAATATCG-3′, which allowed insertion of the gene sequence into the pCDFDuet-1 NcoI restriction site located upstream of the hexa-histidine sequence in the vector, and the reverse primer listed previously. The *csocbbO* (Hneap_0910) gene was amplified via PCR from *H. nea* genomic DNA with forward primer 5′-*GGATCC*GATGAACCCAGCGACTGAA-3′ and reverse primer 5′-*AAGCTT*CTATCGCGTCATCGACAAAT-3′. The amplified gene product was cloned as described previously and was ligated into the BamHI and HindIII sites of the pETDuet-1 vector. Plasmids pETDuet-HTCbbO and pCDFDuet-NTCbbQ were co-transformed into *E. coli* BL21(DE3) chemically competent cells and the transformants grown on LB-agar medium supplemented with ampicillin and spectinomycin. Expression and purification of the complex followed the same procedure described for recombinant CsoCbbQ. The molecular weight of the complex was estimated by size exclusion chromatography (SEC) on a prepacked Superdex 200 10/300 GL column (GE Life Sciences). A protein standard solution of thyroglobulin (669 kDa), apoferritin (443 kDa), β-amylase (200 kDa), alcohol dehydrogenase (150 kDa), and BSA (66 kDa) (Sigma-Aldrich) was used for molecular weight determination; a blue dextran solution was used to determine the column void volume. Runs were performed with the BioLogic DuoFlow System (Bio-Rad). Sample injection volumes were 100 μl and a de-gassed buffer solution of 10 mM Tris-HCl, pH 8.0, with 100 mM NaCl was used as the column eluent at a flow rate of 0.25 ml/min. Absorbance was measured by a BioLogic QuadTec UV-Vis Detector (Bio-Rad). The CsoCbbO:CsoCbbQ ratio of the purified complex was determined by spot densitometry analysis with Quantity One^®^ software in a Bio-Rad 4000 MP Imaging system, following SDS-PAGE separation of peak fractions collected during SEC.

### Bioinformatics

Chemoautotrophs with *cso* operon gene clusters were selected by combining search results from non-redundant homology searches with the Basic Local Alignment Search Tool (BLAST) algorithm and top homolog hits on the Joint Genome Institute Integrated Microbial Genome (JGI-IMG) database, using the α-carboxysome-specific CsoS2 peptide as a diagnostic marker. A Bit Score of 100 was used as a cut-off value. After discarding the α-cyanobacteria, 32 chemoautotrophs were examined for co-occurrence of *cbbO* and *cbbQ* genes using the gene ortholog neighborhood function in the JGI-IMG database. The sequence logo was generated with Weblogo^49^ and the phylogenetic tree was made with PhyML^50^.

### Crystallization and structure determination

CsoCbbQ was crystallized at 22°C at concentrations of 5–10 mg/ml in 0.1 M HEPES pH 7.5, 6% PEG-8000, 8% ethylene glycol in sitting drop trays. Crystals were stabilized by adding 15% ethylene glycol to the crystallization drop before flash freezing in liquid nitrogen. Heavy atom derivatives were obtained by adding 0.1 μl of a 10 mM solution of Thiomersal (Ethyl (2-mercaptobenzoato-(2-)-O,S) mercurate(1-) sodium) to a 10 μl drop containing the stabilized crystals. Diffraction data were collected at beam line 5.0.2 of the Advanced Light Source of the Lawrence Berkeley National Lab. Diffraction data were integrated with XDS^51^ and scaled with SCALA (CCP4)^52^. The structure of CsoCbbQ was solved by locating the mercury modified cysteines using phenix.phaser^53^ and using those sites to generate an initial electron density map. Automatic building of a model into the density using buccaneer^52^ was followed by manual rebuilding/refinement cycles using COOT^54^ and phenix.refine. Statistics for diffraction data collection, structure determination and refinement are summarized in Table 2. Figures of crystal structures were prepared using pymol (www.pymol.org) molecular visualization software.

## Additional Information

**How to cite this article**: Sutter, M. *et al.* Structural Characterization of a Newly Identified Component of α-Carboxysomes: The AAA+ Domain Protein CsoCbbQ. *Sci. Rep.* **5**, 16243; doi: 10.1038/srep16243 (2015).

## Supplementary Material

Supplementary Information

## Figures and Tables

**Table 1:**
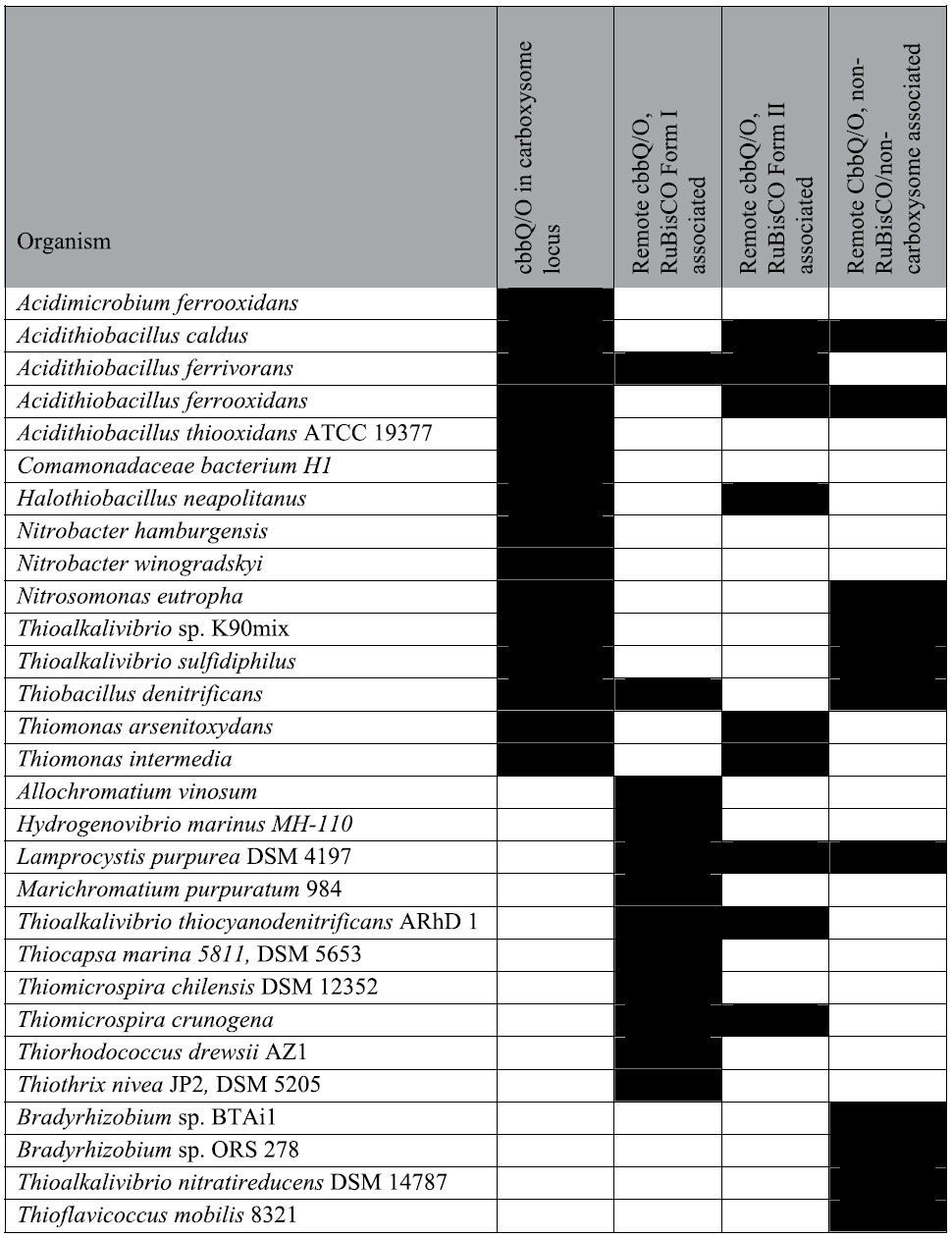
Distribution of *cbbQ* and *cbbO* genes in autotrophic bacteria.

**Table 2:** Data collection and refinement statistics.

	*H. nea*CsoCbbQ
Data collection	
** **Space group	R3:H
****Cell dimensions	
** ***a*, *b*, *c* (Å)	171.9, 171.9, 53.6
** **α, β, γ (°)	90, 90, 120
** **Resolution (Å)	38.8-2.8 (2.9-2.8)*
** ***R*_merge_	0.113 (0.314)
** ***I*/σ*I*	13.0 (4.5)
** **Completeness (%)	99.9 (100.0)
** **Redundancy	4.8 (4.8)
	
Refinement	
** **Resolution (Å)	38.8-2.8
** **No. reflections	14501
** ***R*_work/_ *R*_free_ (%)	22.1 / 28.0
** **No. atoms	3709
** **Protein	3655
** **Ligand/ion	54
** **Water	0
** **B-factors	74.9
** **Protein	75.0
** **Ligand/ion	67.5
** **Water	n/a
** **R.m.s deviations	
** **Bond lengths (Å)	0.004
** **Bond angles (°)	1.00

1 crystal per dataset.

*Highest resolution shell is shown in parentheses.
