# Supplementary material for: Structural Characterization of a Newly Identified Component of α-Carboxysomes: The AAA+ Domain Protein CsoCbbQ
Source: Sci Rep. 2015 Nov 5;5:16243. doi: 10.1038/srep16243 (PMC4633670; doi:10.1038/srep16243)
Supplement: Supplementary Information [file srep16243-s1.pdf]

Supplementary Information
for

Structural Characterization of a Newly Identified Component of α-Carboxysomes: The AAA+ Domain Protein CsoCbbQ

Markus Sutter[1,2], Evan W. Roberts[3], Raul C. Gonzalez[2], Cassandra Bates[3], Salma Dawoud[3], Kimberly Landry[3], Gordon C. Cannon[3], Sabine Heinhorst[3] and Cheryl A. Kerfeld[1,2,4,5*]

[1]MSU-DOE Plant Research Laboratory, Michigan State University, East Lansing, MI 48824, USA
[2]Physical Biosciences Division, Lawrence Berkeley National Laboratory, 1 Cyclotron Road, Berkeley, CA 94720, USA
[3]Department of Chemistry and Biochemistry, The University of Southern Mississippi, 118 College Dr. #5043, Hattiesburg, MS 39406, USA
[4]Department of Plant and Microbial Biology, UC Berkeley, Berkeley, CA 94720, USA
[5]Department of Biochemistry and Molecular Biology, Michigan State University, East Lansing, MI 48824, USA

*To whom correspondence should be addressed: Cheryl A. Kerfeld, +1-510-486-6078 / +1-517-432-4371, ckerfeld@lbl.gov

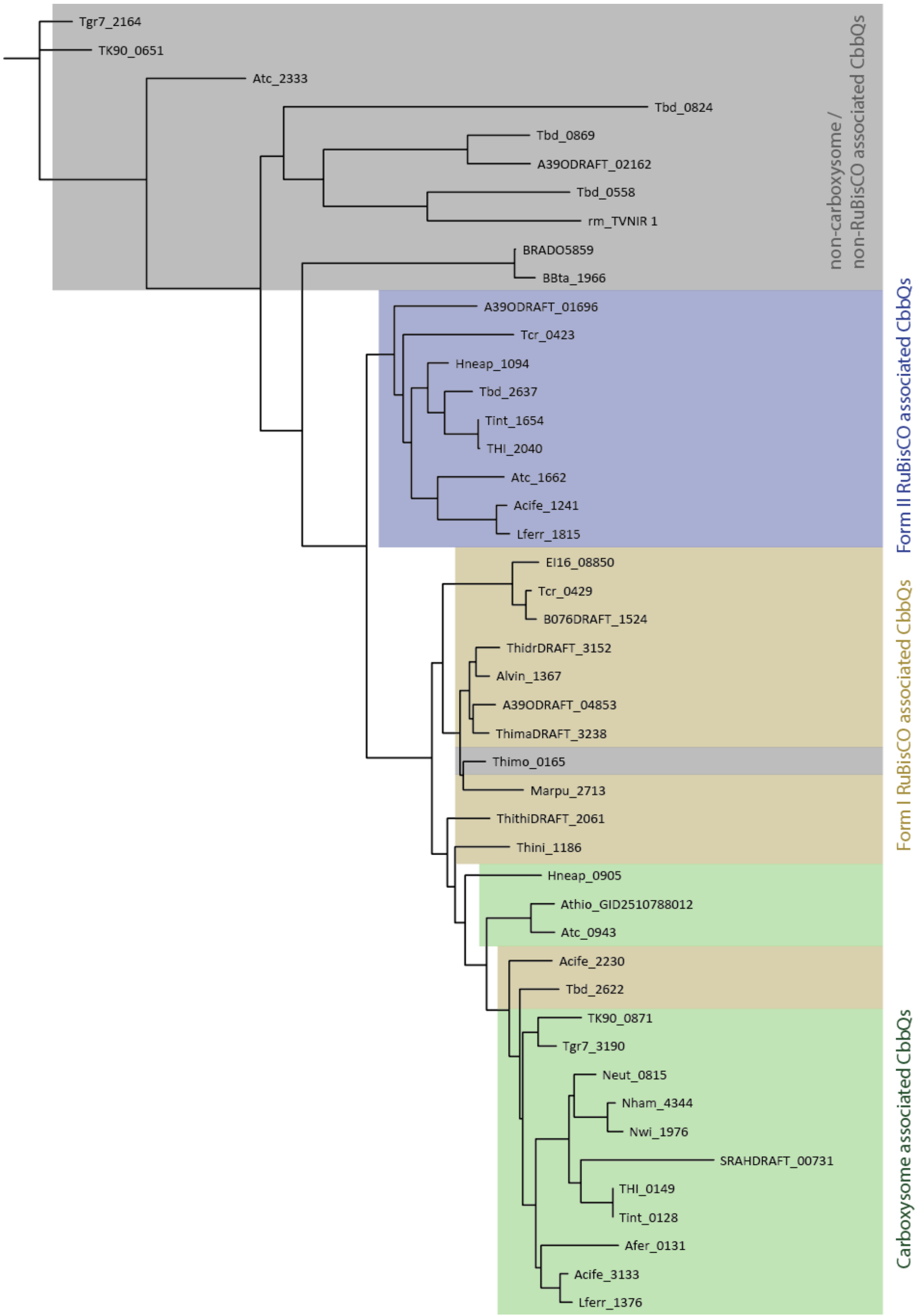

Supplementary Figure 1. Maximum likelihood tree generated using PhyML of selected CbbQ homologs identified by locus tag, with coloring according to genetic context. For full species names see Supplementary Table 1.

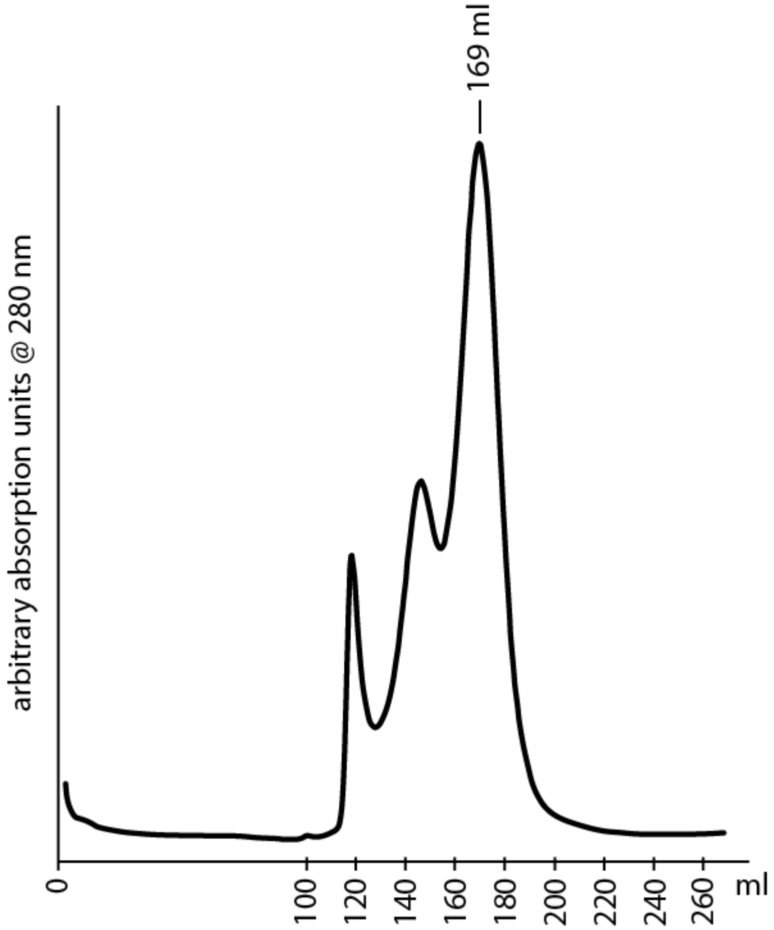

Supplementary Figure 2. Size exclusion chromatography of CsoCbbQ on a Superdex S200 26/60 column. CbbQ was the main component of the largest peak, eluting at 169 ml, corresponding to calculated size of 163 kDa, 5.3 times the calculated molecular weight of a monomer. Standards: Thyroglobulin (670 kDa / 124.2 ml), γ-globulin (158 kDa / 170.1 ml), Ovalbumin (44 kDa / 207.1 ml, Myoglobin (17 kDa / 245.7 ml).

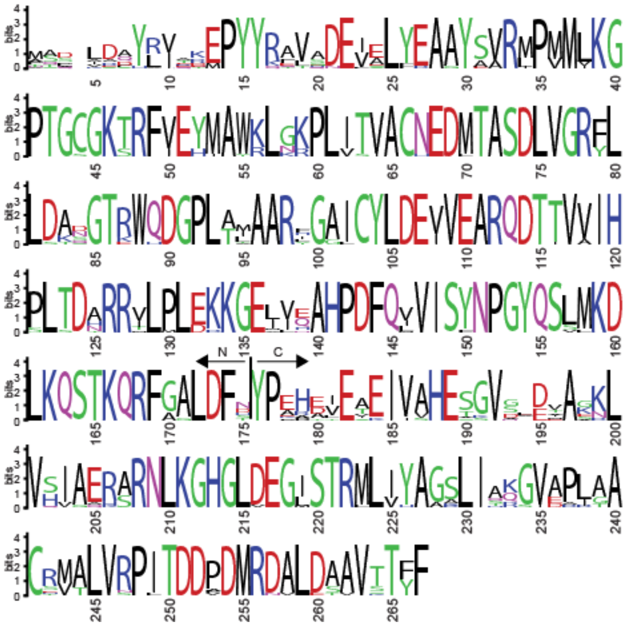

Supplementary Figure 3. Sequence conservation logo of all CbbQ sequences associated with carboxysome loci (residues colored according to property, polar: green, neutral: purple, basic: blue, acidic: red, hydrophobic: black).

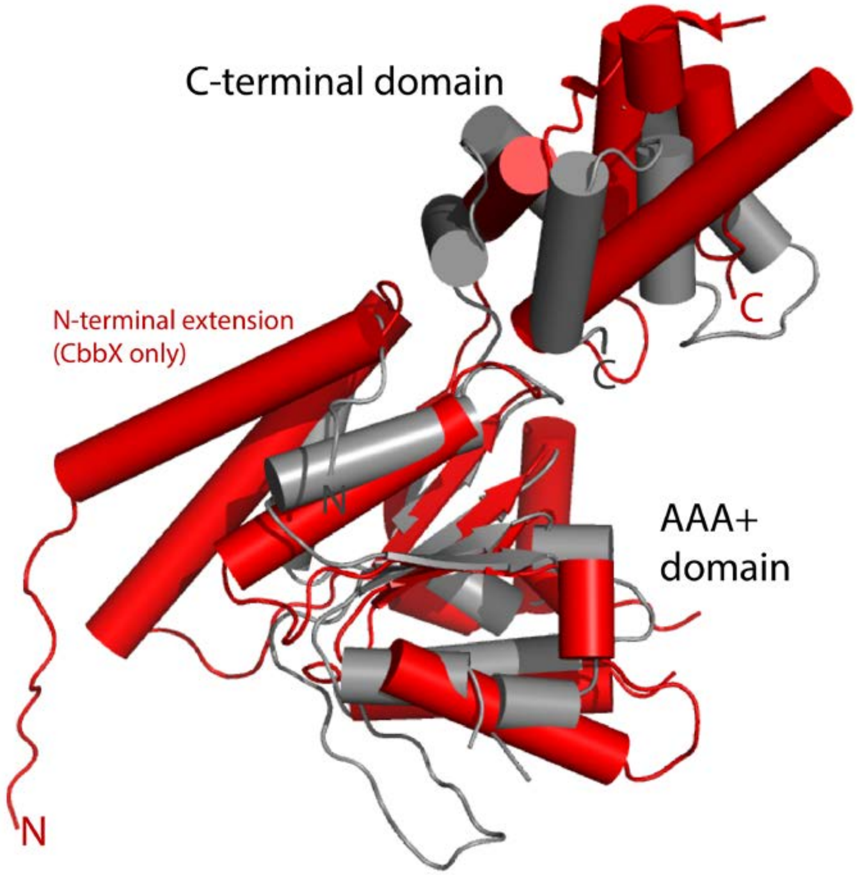

Supplementary Figure 4. Cartoon representation of the structural alignment of CsoCbbQ (grey) and CbbX (red) from *Rhodobacter sphaeroides* (PDB ID 3SYL). There is credible structural alignment of the AAA+ N-terminal α/β subdomain (containing the nucleotide binding motif) of CbbX and CsoCbbQ (α0 to β4 of CsoCbbQ), with an rmsd of 3.8 Å over 79 aligned Cα atoms. However, there is no sensible alignment possible of the C-terminal domains, even when trying to align them without the AAA+ N-terminal α/β subdomains. CbbX also has 2 N-terminal helices which are not present in CsoCbbQ (labeled N-terminal extension).

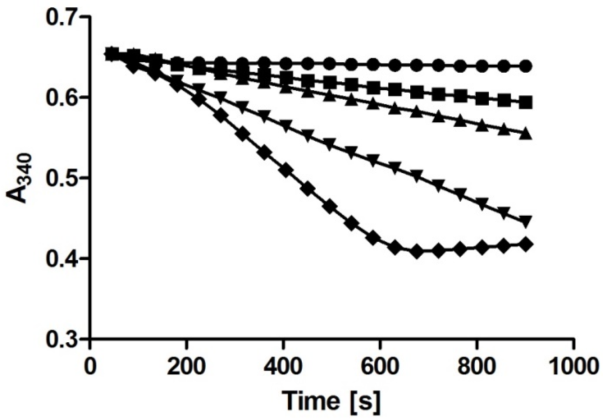

Supplementary Figure 5. NADH-coupled ATPase assay of CsoCbbQ. Runs represent identical assay conditions with CsoCbbQ concentrations of 0 μM (●), 3.7 μM (■), 7.4 μM (▲), 14.8 μM (▼), and 29.6 μM (♦).

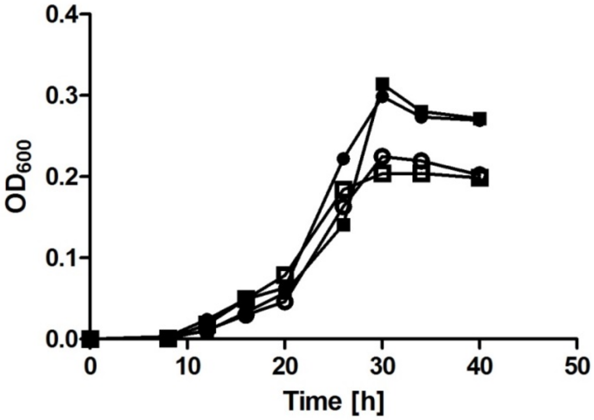

Supplementary Figure 6. Growth curve of 100 ml batch cultures of WT cells in 5% CO_2 (●) and air (○), and *HncbbQ::Km* mutant cells in 5% CO_2 (■) and air (□). Mutant cells reached an equivalent OD as WT at a similar rate.

Supplementary Table 1. Locus tags for cbbQ and cbbO homologs used in this study

	cbbQ in carboxysome operon	cbbO (cbbL/cbbS/csoS2)	remote cbbQ, Rbc form I associated (cbbO gene/cbbL/cbbS)	remote cbbQ, Rbc form II associated (cbbO gene/cbbL)	remote, non-Rbc associated (cbbO gene)
Acidimicrobium ferrooxidans	Afer_0131	Afer_0132 (0119/0120/0121)			
Acidithiobacillus caldus	Atc_0943	Atc_0938 (0926/0927/0928)		Atc_1662 (1663/1661)	Atc_2333 (2332)
Acidithiobacillus ferrivorans	Acife_3133	Acife_3132 (3145/3144/3143)	Acife_2230 (2229/2232/2231)	Acife_1241 (1240/1242)	
Acidithiobacillus ferrooxidans	Lferr_1376	Lferr_1375 (1389/1388/1387)		Lferr_1815 (1816/1814)	Lferr_2164(2163)
Acidithiobacillus thiooxidans ATCC 19377	GID2510788012	GID2510788007 (2510787994/5/6)			
Comamonadaceae bacterium H1	SRAHDRAFT_00731	SRAHDRAFT_00732 (00720/00721/00722)			
Halothiobacillus neapolitanus	Hneap_0905	Hneap_0910 (0922/0921/0920)		Hneap_1094 (1092/1095)	
Nitrobacter hamburgensis	Nham_4344	Nham_4345 (4332/4333/4334)			
Nitrobacter winogradskyi	Nwi_1976	Nwi_1975 (1987/1986/1985)			
Nitrosomonas eutropha	Neut_0815	Neut_0816 (0804/0805/0806)			Neut_0519 (0518)
Thioalkalivibrio sp. K90mix	TK90_0871	TK90_0872 (0858/0859/0860)			TK90_0651 (0652)
Thioalkalivibrio sulfidiphilus	Tgr7_3190	Tgr7_3189 (3203/3202/3201)			Tgr7_2164 (2163)
Thiomonas arsenitoxydans	THI_0149	THI_0150 (0135/0136/0137)		THI_2040 (2039/2041)	
Thiomonas intermedia	Tint_0128	Tint_0129 (0115/0116/0117)		Tint_1654 (1655/1653)	
Allochromatium vinosum			Alvin_1367 (1368/1365/1366)		
Hydrogenovibrio marinus MH-110			EI16_08850 (08855/08840/08845*)		
Lamprocystis purpurea DSM 4197			A39ODRAFT_04853 (04854/04851/04852)	A39ODRAFT_01696 (none/01697)	A39ODRAFT_02162 (02165)
Marichromatium purpuratum 984			Marpu_2713 (2715/2711/2712)		
Thioalkalivibrio thiocyanodenitrificans ARhD 1			ThithiDRAFT_2061 (2062/2059/2060)		
Thiocapsa marina 5811, DSM 5653			ThimaDRAFT_3238 (3240/3236/3237)		
Thiomicrospira chilensis DSM 12352			B076DRAFT_1524 (1523/1526/1525)		
Thiomicrospira crunogena			Tcr_0429 (0430/0427/0428)	Tcr_0423 (0422/0424)	
Thiobacillus denitrificans			Tbd_2622 (2621/2624/2623)	Tbd_2636 (2636/2638)	Tbd_0869 (0868), Tbd_0558(0555), Tbd_0824(0825)
Thiorhodococcus drewsii AZ1			ThidrDRAFT_3152 (3154/3150/3151)		
Thiothrix nivea JP2, DSM 5205			Thini_1186 (1187/1183/1184)		
Bradyrhizobium sp. BTAi1					BBta_1966 (1965)
Bradyrhizobium sp. ORS 278					BRADO5859 (BRADO5860)
Thioalkalivibrio nitratireducens DSM 14787					TVNIR_1657 (1658)
Thioflavicoccus mobilis 8321					Thimo_0165 (0167)

**consecutive genes 5 numbers apart*
